# Factors associated with critical care requirements in diabetic patients treated with dexamethasone for COVID-19 infection in the first wave of the pandemia

**DOI:** 10.3389/fendo.2022.1009028

**Published:** 2022-12-22

**Authors:** Sol Batule, Berta Soldevila, Carme Figueredo, María Teresa Julián, Laia Egea-Cortés, Juliana Reyes-Ureña, Jordi Casabona, Lourdes Mateu, Roger Paredes, Bonaventura Clotet, Rosa López, Manel Puig-Domingo, Núria Alonso

**Affiliations:** ^1^ Department of Endocrinology and Nutrition, Germans Trias i Pujol Research Institute and Hospital, Badalona, Spain; ^2^ Department of Medicine, Universitat Autònoma de Barcelona, Barcelona, Spain; ^3^ Centre d'Estudis Epidemiològics sobre les Infeccions de Transmissió Sexual i Sida de Catalunya (CEEISCAT), Badalona, Spain; ^4^ Infectious Disease Service, Germans Trias i Pujol Research Institute and Hospital, Badalona, Spain; ^5^ IrsiCaixa AIDS Research Institute, Hospital Germans Trias i Pujol, Badalona, Spain; ^6^ Direcció d'Organització i Sistemes Gerència Territorial Metropolitana Nord, Institut Català de la Salut, Badalona, Spain

**Keywords:** diabetes, hyperglycemia, COVID-19, dexamethasone, SARS-CoV-2

## Abstract

**Introduction:**

Diabetes mellitus (DM) and hyperglycemia are important risk factors for poor outcomes in hospitalized patients with coronavirus disease 2019 (COVID-19). The aim of the present study was to analyze the factors associated with the composite outcome of the necessity of invasive mechanical ventilation (IMV) or admission to the intensive care unit (ICU) in subjects with severe COVID-19 infection treated with dexamethasone comparing patients with DM vs. patients without DM.

**Research design and methods:**

An observational retrospective cohort study was performed, including hospitalized subjects with a diagnosis of SARS-CoV-2 pneumonia. Inclusion criteria were: age ≥18 years old with severe COVID-19 disease requiring daily intravenous 6 mg dexamethasone treatment for 10 days. Exclusion criteria were: <18 years old, non-severe illness and/or patients in charge of ICU. Variables related to clinical and analytical parameters, glycemic control, acquired-hospital superinfections, mortality, IMV requirement, ICU admission and length of stay were included.

**Results:**

Two hundred and nine individuals with COVID-19 disease treated with dexamethasone were included. One hundred twenty-five out of these subjects (59.8%) were patients with DM. Overall, from the 209 subjects, 66 (31.6%) required IMV or were admitted to the ICU, with significant differences between patients with DM (n=50) vs. patients without DM (n=16) (76% vs. 24%, p=0.002). Among the group of subjects with DM (n=125), those who required IMV or were admitted to the ICU showed higher serum concentrations of C-reactive protein, interleukin-6, D-dimer, ferritin and pro-calcitonin and significantly lower serum concentrations of albumin compared to those who did not require IMV or were not admitted to the ICU. Besides, between these two groups of patients with DM, we observed no differences in glycemic parameters, including median capillary blood glucose values, glycosylated hemoglobin, coefficient of variability and hypoglycemic episodes. In the multinomial analysis, factors independently associated with the composite outcome of IMV or admission to the ICU in the insulin-treated group were the National Early Warning Score (NEWS) 2 score (OR 1.55 [1.17-2.17], p=0.005) and the presence of hospital-acquired superinfections (OR 35.21 [5.11-386.99], p=0.001).

**Conclusions:**

In our study, parameters related to glycemic control were not associated with IMV requirement nor admission to the ICU in patients with DM and severe COVID-19 disease receiving daily 6 mg of dexamethasone for 10 days. However, hospital-acquired superinfections and disease severity at admission were independent factors associated with this composite outcome.

## Introduction

Type 2 Diabetes Mellitus (T2D) is the second most frequent comorbidity after arterial hypertension in patients admitted for severe acute respiratory syndrome coronavirus 2 (SARS-CoV-2), the virus that causes coronavirus disease 2019 (COVID-19) (1). It is now well-established that T2D is a risk factor associated with COVID-19 disease severity, with meta-analyses indicating that pre-existing diabetes mellitus (DM) is associated with a greater risk of severe disease and death with an odds ratio ranging from 1.9 to 2.68 (2), especially in those cases with a poor blood glucose control during hospitalization (3). This risk has been reported to be increased both in subjects with type 1 diabetes mellitus (T1D) and T2D (4, 5). On the other hand, COVID-19 might also predispose infected individuals to the development of hyperglycemia. This can be due to the enhanced viral-induced inflammation, in particular, interleukin 6 (IL-6) mediated, which increases insulin resistance in a dose-dependent manner (6). Indeed, the so-called ‘cytokines storm’ scenario resulting from severe COVID-19 is associated not only with an enhancement of insulin-resistance situation but also with impaired insulin production from the pancreatic β cells (7). Several studies have shown that uncontrolled inpatient hyperglycemia is associated with adverse outcomes, including increased rates of infection and mortality and longer hospital length of stay (LOS) (8–10).

A frustrating challenge in the COVID-19 pandemic has been the lack of effective treatments, particularly those of viricide effect (11) during the first waves. At the time of writing this article, different new therapeutic approaches are available and vaccination has been performed in most of the developed countries. Pre-exposure prophylaxis with monoclonal antibodies in severely immunocompromised individuals; early treatments in outpatients with mild-moderate COVID-19 at high risk for progression to severe disease with Nirmatrelvir/Ritonavir, Remdesivir or monoclonal antibodies; and finally treatment of hospitalized patients with moderate-severe COVID-19 with Remdesivir, steroids, Tocilizumab or Barcitinib are currently available. Furthermore, the fourth dose of the vaccine will be administered to patients at risk (12). However, when this study was carried out, we only had Remdesivir and steroids in hospitalized patients with moderate-severe COVID-19 and the vaccination campaign just started in December 2020. During the first waves of the pandemic, no pharmacological intervention for COVID-19 disease has been as successful as steroids for treating acute illness aiming at minimizing the cytokine storm effects. The RECOVERY (Randomised Evaluation of COVID‐19 Therapy) trial, found that dexamethasone at a dose of 6 mg once daily for up to 10 days reduces mortality by one‐third in ventilated patients and by one‐fifth in other patients, receiving oxygen therapy (13). Following these results, the use of short courses of high doses of glucocorticoid therapy has been widely used. The benefits of dexamethasone for patients with COVID-19 likely arise from its immunosuppressive and anti-inflammatory properties. Indeed, some of the most potent anti-inflammatory actions of glucocorticoids occur by inhibiting the production of pro-inflammatory cytokines (IL-1, TNF and IL-6) (14). However, it is known that high doses of glucocorticoids also exacerbate hyperglycemia in people with DM, may unmask previously undiagnosed DM and, in those at risk of DM development, may precipitate hyperglycemia and new-onset DM. It has to be mentioned that no data are available in the RECOVERY trial regarding the occurrence of hyperglycemia at different times during and after the use of dexamethasone, nor in the impact that the use of corticosteroids may have on glycemic control. Hyperglycemia, a common side effect of corticosteroids that usually requires insulin treatment, is associated with worse outcomes in COVID-19 (15, 16).

To our knowledge, inpatient glycemic control in COVID-19 infected subjects treated with dexamethasone and its related outcomes has not been well documented in previous studies. Thus, the aim of the present study was to analyze the factors, among them glycemic control, associated with the composite outcome of invasive mechanical ventilation (IMV) or admission to intensive care unit (ICU) and LOS, in patients with COVID-19 treated with dexamethasone comparing patients with DM vs. patients without DM. Also, the secondary endpoint was to analyze, in the group of patients treated with insulin, whether there are any differences between patients who have a long LOS, mortality, or hospital-acquired superinfections vs. those who do not.

## Materials and methods

### Study design and setting

We performed an observational retrospective cohort study, including individuals with a diagnosis of SARS-CoV-2 pneumonia according to the World Health Organization (WHO) guidelines (17) and admitted to the conventional ward in charge of the Infectious Diseases Department from October 2020 to January 2021.

Data were obtained from an anonymized electronic health records database from the Germans Trias i Pujol University Hospital (HUGTiP) (Badalona, Spain). The database included retrospective clinical information, information on admission, diagnostic and procedure codes, prescribed medications, and laboratory parameters. All patients included in the study were followed up during the LOS from the start of dexamethasone treatment until discharge, ICU requirement or death, whichever occurred first.

The study was approved by the Ethics Committee of the HUGTiP (approval n° PI-20-175).

### Inclusion and exclusion criteria

Inclusion criteria were: age ≥18 years old with severe COVID-19 disease requiring daily intravenous 6 mg dexamethasone treatment for 10 days. Dexamethasone was implemented according to the Catalonia Department of Health protocol based on Infectious Diseases Society of America guidelines (18) and the RECOVERY study (13). Severe COVID-19 disease was defined as when patients presented with SpO2 ≤94% on room air, including those on supplemental oxygen. In non-severe illness defined as SpO2 >94% not requiring supplemental oxygen, dexamethasone treatment was not indicated. Exclusion criteria were: <18 years old, non-severe illness and/or patients in charge of ICU and/or IVM requirement.

### Study variables

The following baseline variables were collected: a) demographic and anthropometric data: ethnicity, sex, age and body mass index (BMI); b) main pathological history: previously known DM, hypertension, dyslipidemia, cardiovascular disease (defined as myocardial infarction and/or heart failure), chronic obstructive pulmonary disease and chronic kidney disease (defined as estimated glomerular filtration rate (eGFR) lower than 60 ml/min/1.73m2) and c) outpatient regular antidiabetic treatment.

Severity on admission was graded according to the National Early Warning Score (NEWS) 2 scale (19) in which a score is allocated to six physiological parameters: respiratory rate, oxygen saturation, systolic blood pressure, pulse rate, temperature and level of consciousness. Each parameter scores from 0 to 3 and an additional two points are added if the patient is receiving oxygen therapy. The total possible score ranges from 0 to 20 and, the higher the score the greater the clinical risk meaning the need for escalation, possible clinical intervention and more intensive monitoring.

The presence of acquired-hospital superinfections (infections diagnosed >48 hours after admission), including sepsis, urinary tract infection, bacterial respiratory infection and mycosis, was recorded. We also analyzed the LOS and determined that a long LOS was more than 10 days from admission until discharge or death.

Blood laboratory variables were collected at admission: hemogram including leukocytes, lymphocytes and neutrophils recount; liver and renal function including aspartate aminotransferase (AST), alanine transaminase (ALT), gamma-glutamyl transferase (GGT), eGFR; lipid function including total cholesterol, LDL-c, HDL-c, triglycerides; inflammatory parameters including fibrinogen, troponin, D-dimer, ferritin, IL-6, C-reactive protein (CRP), pro-calcitonin and albumin.

Assessment of metabolic status included blood glucose at admission before starting dexamethasone treatment and HbA1c measured within 48 hours of admission if the patient did not have a determination in the last three months.

Regarding glycemic control, we defined patients with diabetes as history of DM or use of any antidiabetic medication before hospitalization, newly diagnosed DM (fasting glucose glucose ≥126 mg/dL or HbA1c ≥ 6.5% and no previous diagnosis of DM), prediabetes (HbA1c 5.7-6.4%) and glucocorticoid-induced hyperglycemia (HbA1c ≤ 5.6% but fasting glucose ≥126 mg/dL in more than one consecutive measure). Patients with normal glucose (fasting glucose concentrations below 126 mg/dL or capillary blood glucose measurement (CBGM) before main meals <180 mg/dL) were considered subjects without diabetes.

For patients with previously known DM, insulin treatment was initiated at admission. In these patients, CBGM was performed according to the HUGTiP protocol, four times per day before main meals and bedtime. For patients without a previously known DM diagnosis, CBGM was performed three times per day, before main meals since the start of dexamethasone treatment and, if glycemia was ≥180 mg/dL in two consecutive measures, insulin was initiated. In patients with CBGM <180 and fasting blood glucose (FBG) <126 mg/dL during the dexamethasone treatment period, insulin treatment was not indicated. Values of CBGM were recorded for further analysis only in the group of patients who required insulin and during the period of glucocorticoid treatment. According to CBGM values, mean CBGM, standard deviation (SD) of CBGM and glycemic coefficient of variability (CV) were assessed.

Mean CBGM was calculated as the mean of all CBGM per day. The standard deviation of CBGM was calculated as the SD of the means of all CBGM measurements per day. Glycemic CV was calculated as SD of CBGM concentration/mean CBGM x100 of all CBGM. We defined optimal glycemic control as a median CBGM ≤180 mg/dL and suboptimal glycemic control as a median CBGM >180 mg/dL. Hypoglycemic episodes were also assessed and were defined as grade 1 (54-70 mg/dL) and grade 2 (<54 mg/dL).

We established a revised hospital protocol for the treatment of hyperglycemia in COVID-19 patients who were treated for 10 days with an intravenous dexamethasone dose of 6 mg per day. According to this protocol, the initial total dose of insulin ranged from 0.5 up to 1.2 UI/Kg/d taking into account previous diagnosis of DM, previous antidiabetic therapy and glycemic control at admission (Appendix A).

### Statistical analysis

Quantitative variables were summarized according to their distribution [median, interquartile range or mean and standard deviation ( ± SD)] and categorical variables as frequency, number and percent (%). A bivariate analysis was carried out using the Mann-Whitney U test to compare medians and chi-square tests to compare categorical differences.

Multinomial logistic regression analyses were used to identify variables associated with the composite of IMV or admission to the ICU and with the LOS in subjects treated with insulin. To reach the optimal model we use the Deletion/Substitution/Addition (DSA) algorithm, which is an iterative process where the final model is selected by minimizing the residual mean squared error (RMSE) using 5-fold cross-validation. As this method is based on cross-validation (random method), we ran the DSA 50 times to make the results more consistent and to keep the exposures that appear at least 5% of the time. We fixed age, gender, BMI and NEWS score as potential confounding variables.

## Results

**Figure 1:**
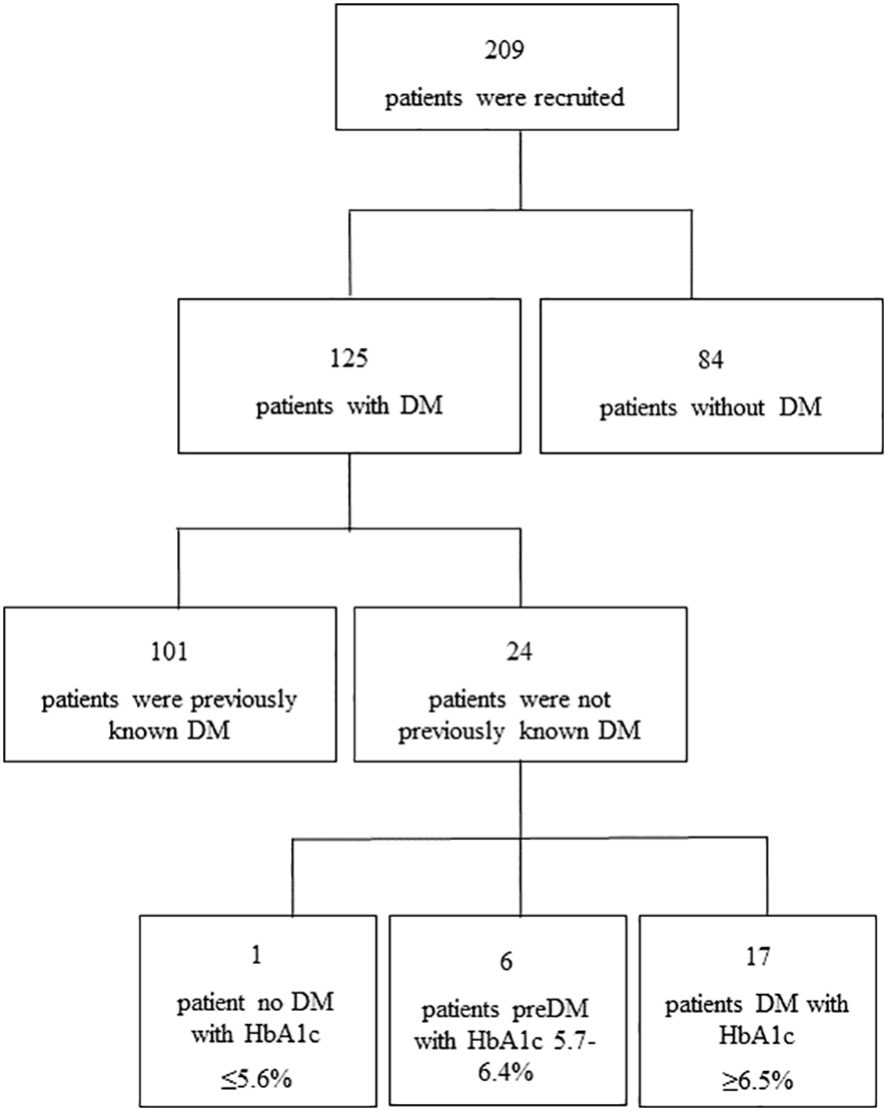
Flowchart of study population.

Two hundred and nine individuals with COVID-19 treated with dexamethasone were included. One hundred twenty-five out of these subjects were patients with DM and required insulin treatment during their hospital stay. Of these 125 subjects, 101 presented DM previously known, 17 presented an unknown DM with HbA1c value ≥ 6.5%, 6 had prediabetes (HbA1c 5.7-6.4%) and only 1 subject had glucocorticoid-induced hyperglycemia (HbA1c ≤ 5.6%) (**Figure 1**).

Subjects with DM were older (median 72 [39.2-90] vs. 59.5 [27.15-89.92] years, p<0.001), with a higher BMI (median 30.1 [22.67-46.73] vs. 28.98 [20.89-37.75] kg/m², p=0.02) and presented more associated comorbidities such as cardiovascular disease (38.4 vs. 19.05%, p=0.005), hypertension (69.6 vs. 30.95%, p<0.001) and dyslipidemia (62.4 vs. 44.05%, p=0.013) compared with subjects without DM (**Table 1**). Patients with DM also showed a higher NEWS2 score at hospital admission (median 5 [0-10.9] vs. 3. [0-8] points, p<0.001). Regarding biochemical parameters, subjects with DM showed significantly higher HbA1c values (median 7.45 [5.9-12.2] vs. 5.4 [5.2-5.5] %, p<0.001), higher admission blood glucose (median 156 [114-289] vs. 102 [96-106] mg/dL, p<0.001) and lower serum levels of albumin compared with those who did not require insulin treatment (median 35.1 [27.52-42.97] vs. 39.65 [23.15-43.75] g/L, p=0.01). No differences were observed in inflammatory parameters, including CRP, pro-calcitonin, ferritin, IL-6 or fibrinogen, or D-dimer levels between the two groups (**Table 1**).

**Table 1:** Baseline clinical characteristics of patients with COVID-19 treated with dexamethasone.

	Patients without DM	Patients with DM	P-value
**Patients n(%)**	84 (40.19)	125 (69.81)	
Age (years)*	59.5 (27.2-90)	72 (39.2-90)	<0.001
**Sex (F) n(%)**	34 (40.5)	59 (47.2)	0.414
BMI (Kg/m²)*	28.98 (20.9-37.8)	30.1 (22.7-46.7)	0.02
**Medical History**			
Diabetes mellitus, n(%)	–	101 (80.8-9)	
Cardiovascular disease, n(%)	16 (19.1)	48 (38.4)	0.005
Chronic kidney disease, n(%)	12 (14.3)	32 (25.6)	0.411
Hypertension, n(%)	26 (31)	87 (69.6)	<0.001
Dyslipidemia, n(%)	37 (44.1)	78 (62.4)	0.013
NEWS2 score*	3 (0-8)	5 (0-11)	<0.001
**Remdesivir treatment, n(%)**	35 (41.7)	48 (38.4)	0.777
Glycemic parameters*			
Admission plasmatic glucose (mg/dL)	102 (96-106)	156 (114-289)	<0.001
HbA1c (%)	5.4 (5.2-5.5)	7.45 (5.9-12.2)	<0.001
Biochemical markers*			
Leukocytes count (x10^9^/L)	6.2(3.2-20.6)	7 (2.9-13.3)	0.336
Neutrophil count (x10^9^/L)	4.4 (1.9-17.5)	4.7 (1.7-12)	0.372
Lymphocyte count (x10^9^/L)	1 (0.3-2.19)	0.9 (0.3-2.5)	0.793
CRP (mg/L)	58.8 (6.42-283.9)	75.9 (5.2-287.7)	0.485
Pro-calcitonin (ng/mL)	0.1 (0-1.3)	0.1 (0-2)	0.131
D-Dimer (ng/mL)	830 (238.3-20572.6)	808.5 (195.1-7597.8)	0.844
Troponin (pg/mL)	6.4 (0-108.9)	8.3 (0.4-123.9)	0.045
Ferritin (ng/mL)	460 (34.8-3965.5)	415.5 (26.4-1864.3)	0.287
IL-6 (pg/mL)	37.8 (4.8-202)	39.8 (1.68-244.4)	0.56
Fibrinogen	727 (426.8-1008.7)	708 (376-1049.1)	0.366
Albumin (g/L)	37.7 (23.2-43.8)	35.1 (27.5-43)	0.01
AST (U/L)	29.5 (15-104.6)	27 (11-129.3)	0.821
ALT (U/L)	30.5 (9.7-131.2)	25 (8.1-146.6)	0.1
GGT (U/L)	44 (10.2-368.3)	49 (15.8-600.8)	0.926
Cholesterol (mg/dL)	168 (68-232.4)	146.5 (54.5-237.4)	0.323
HDL-c (mg/dL)	46.3 (24.5-71.5)	38.1 (20.2-75)	0.049
LDL-c (mg/dL)	122.5 (34.1-187.5)	85.5 (20.5-165.3)	0.011
Triglycerides (mg/dL)	161.5 (47-437.1)	137 (46.8-387.1)	0.552
eGFR (ml/min/1.73m^2)^	90 (20.3-90)	87 (21.2-90)	0.156
**Outcomes**			
IMV requirement/ICU admission, n(%)	16 (19.1)	50 (40)	0.002
Length of stay, days*	9 (3-28)	13 (4-46)	<0.001
Mortality, n(%)	4 (4.8)	15 (12)	0.124
Superinfections n (%)	10 (11.9)	16 (12.8)	1

Data are median (IQR95%). DM, diabetes mellitus; BMI, body mass index; CRP, C-reactive protein; IL-6, interleukin 6; AST, aspartate aminotransferase; ALT, alanine transaminase; GGT, gamma-glutamyl transferase; eGFR, estimated glomerular filtration rate.

### Composite outcome of IMV requirement or admission to the ICU

From the 209 patients receiving dexamethasone treatment, 66 (31.5%) required IMV or were admitted to the ICU, with a significantly higher percentage in those with DM (n=50) compared to those without DM (n=16) (76% vs. 24%, p=0.002) (**Table 1**).

Among the group of 125 patients with DM, those who required IMV or were admitted to the ICU (n=50) compared with those who did not (n=75), showed a significantly higher NEWS2 score (median 6 [2-13] vs. 4 [0-9] points, p<0.001), serum concentrations of AST (median 35 [13.4-233.2] vs. 25.5 [10.68-96.83] U/L, p=0.021), CRP (median 108.95 [7.92-317.33] vs. 60.7 [4.64-211.95] mg/L, p=0.003), IL-6 (median 49,78 [3.93-778.67] vs. 29.98 [1.58-92.08] pg/mL, p=0.005), D-dimer (median 1071 [210.6-7232] vs. 648 [193.8-5469.6] ng/mL, p=0.018), ferritin (median 547 [90.8-1914.8] vs. 344 [22-1502.4] ng/mL, p=0.009) and pro-calcitonin (median 0.16 [0.02-3.28] vs. 0.1 [0.02-0.5] ng/mL, p=0.008) and significantly lower serum concentrations of albumin (median 33.75 [26.92-42.39] vs. 36 [29.47-43.12] g/L, p=0.002) (**Table 2**). Furthermore, in the group of subjects with DM, no differences were observed in glycemic parameters, such as plasmatic glycemic levels at admission, median CBGM values, SD of CBGM, HbA1c, CV and hypoglycemic episodes in both grade 1 and grade 2, between those who required IVM or admission to the ICU compared with those who did not (**Table 2**). However, the mean daily dose of insulin per kilogram (IU/Kg) of body weight during the follow-up was significantly higher in those who required IMV or admission to the ICU compared with those who did not (median 0.58 [0.31-0.93] vs. 0.48 [0.15-1.15] IU/Kg, p=0.039). No differences were observed in IMV requirement or ICU admission between subjects with optimal glycemic control vs. those with suboptimal glycemic control.

**Table 2:** Baseline clinical characteristics and glycemic parameters in patients with diabetes mellitus related to IMV requirement or admission to ICU.

	Patients who did not require IMV nor were admitted to ICU	Patients who required IMV or were admitted to ICU	P-value
**Patients n(%)**	75 (60)	50 (40)	
Age (years)*	75 (32.9-92.3)	70 (45-83.8)	0.046
**Sex (F/M), n**	39/36	20/30	0.257
BMI (Kg/m²)*	30.1 (22.5-45.5)	30 (23.8-45.3)	0.784
**Medical History**			
Diabetes mellitus, n(%)	60 (80)	41 (82)	0.963
Cardiovascular disease, n(%)	35 (46.7)	13 (26)	0.032
Chronic kidney disease, n(%)	20 (26.7)	12 (24)	0.917
Hypertension, n(%)	52 (69.3)	35 (70)	1
Dyslipidemia, n(%)	47 (62.7)	31 (62)	1
**Glycemic parameters**			
CBG (mg/dL)*	204 (123-317)	207 (129-283)	0.672
SD of CBG (mg/dL)*	68 (32-104)	60 (27-88)	0.22
HbA1c (%)*	7.05 (6-12.2)	7.8 (5.7-10.3)	0.146
CV (%)*	32.3 (19.2-46.2)	28.4 (12.1-44.7)	0.344
Total dose of insulin (units/Kg)*	0.48 (0.15-1.15)	0.58 (0.31-0.93)	0.039
Grade 1 hypoglycemia (episodes)	12	1	0.727
Grade 2 hypoglycemia (episodes)	13	1	0.871
Biochemical markers*			
Leukocytes count (x10^9^/L)	6.9(2.9-12.7)	7.2 (2.9-13.8)	0.12
Neutrophil count (x10^9^/L)	4.5 (1.6-10.2)	5.4 (2-13.9)	0.162
Lymphocyte count (x10^9^/L)	1 (0.3-2.4)	0.8 (0.3-2.9)	0.167
CRP (mg/L)	60.7 (4.6-212)	109 (7.9-317.3)	0.003
Pro-calcitonin (ng/mL)	0.1 (0-0.5)	0.2 (0-3.3)	0.008
D-Dimer (ng/mL)	648 (194-5469.6)	1071 (210.6-7232)	0.018
Troponin (pg/mL)	8.6 (0.7-115.7)	7.9 (0.1-372.3)	0.947
Ferritin (ng/mL)	344 (22-1502.4)	547 (90.8-1914.8)	0.009
IL-6 (pg/mL)	30 (1.6-92.1)	49.8 (3.9-778.7)	0.005
Fibrinogen	682 (382.4-1055.2)	731.5 (380.9-1024.7)	0.14
Albumin (g/L)	36 (29.5-43.1)	33.8 (26.9-42.4)	0.002
AST (U/L)	25.5 (10.7-96.8)	35 (13.4-233.2)	0.021
ALT (U/L)	22 (8.9-149.8)	32.5 (8.7-86.7)	0.075
GGT (U/L)	50 (15-411.5)	46.5 (17.2-615.3)	0.539
Cholesterol (mg/dL)	148.5 (8.3-235.6)	144.5 (64.1-239.4)	0.749
HDL-c (mg/dL)	38.1 (23.9-106.8)	37.8 (19.1-71.2)	0.714
LDL-c (mg/dL)	80 (23.1-171)	91 (23-156)	0.39
Triglycerides (mg/dL)	144 (55.3-450.3)	132.5 (43.8-234.2)	0.239
eGFR (ml/min/1.73m^2^)	85.5 (20-90)	90 (27.4-90)	0.245

*Data are median (IQR95%). BMI, body mass index; CBG, capillary blood glucose; CV, coefficient of variability; TDI, total daily insulin dose; CRP, C-reactive protein; IL-6, interleukin 6; AST, aspartate aminotransferase; ALT, alanine transaminase; GGT, gamma-glutamyl transferase; eGFR, estimated glomerular filtration rate.

Factors independently associated with the composite of IMV or admission to the ICU in the multinomial analysis were the NEWS2 score (OR 1.55 [1.17-2.17], p=0.005) and the presence of hospital-acquired superinfections (OR 35.21 [5.11-386.99], p=0.001). No variables associated with glycemic control were found as independent factors associated with this outcome.

### Overall in-hospital mortality

The overall in-hospital mortality was 9.09% (19 patients out of 209). This mortality rate was higher (12% vs. 4.8%) among patients with DM (n=15) compared with those without DM (n=4) but not statistically significant (**Table 1**).

Among the subjects with DM, those who died were older (median 84 [67-94] vs. 71 [37-90] years, p<0.001), showed significantly higher AST (median 52.5 [17.65-101.9] vs. 26 [11-151.35] U/L, p=0.009) and D-Dimer serum concentrations (median 1764.5 [516.38-3550.7] vs. 734 [194.3-8262.9] ng/mL, p=0.002) compared with those who lived. No differences were observed between dead vs. alive patients in CBGM, SD of CBGM, CV, HbA1c, plasmatic glycemic concentrations at admission and mean daily dose of insulin during the follow-up. Finally, no differences in mortality were observed between patients with DM with optimal glycemic control vs. those with suboptimal glycemic control.

In order to enhance the statistical power of the comparison, we have pooled mortality together with the composite outcome of IMV requirement or admission to the ICU. We found that patients who required ICU/IMV or died (n=58) presented a significantly longer LOS (median 19 [6-31] vs. 11 [6-16] days, p<0.001), higher NEWS2 (median 6 [3-9] vs. 4 [2-6] points, p<0.001), higher leukocytes (median 8.5 [2.3-14.7] vs. 6.5 [3.8-9.1] x10^9^/L, p=0.013), AST (median 49 [39-101] vs. 30 [10-49] U/L, p=0.007) and RCP (median 117.19 [32.28-202.1] vs. 69.93 [11.67-128.19] mg/L, p=0.001) serum concentrations and lower albumin (median 34.03 [29.68-38.38] vs. 36.72 [32.54-40.9] g/L, p=0.001) serum concentrations compared with patients who did not require ICU/IMV and stayed alive (n=67). We found no significant differences regarding glycemic parameters between both groups.

### Length of stay

Length of hospital stay was significantly longer in patients with DM compared with patients without DM (median 13 [IQR 4-45.5) vs. 9 [IQR 3.08-27.7] days, p<0.001) (**Table 1**).

Among those with DM, no differences were observed in LOS between optimal vs. suboptimal controlled patients. Also, we found no statistical differences between long vs. short LOS in median CBGM, SD of CBGM, CV, plasmatic glycemic levels at admission and total daily insulin dose per kg. Statistically significant associations were found regarding higher AST (median 28.5 [13-193.38] vs. 24 [10-53] IU/L, p=0.035) and D-Dimer serum concentrations (median 980 [204.75-5664.5] vs 568 [189.3-9281.85] ng/mL, p=0.037) and higher prevalence of superinfections (12.8% vs 0%, p=0.001) between subjects with long vs. short LOS. In the multinomial analysis, superinfections were associated with a longer LOS (p<0.001).

### Acquired-hospital superinfections

Superinfections were not different between patients with vs. patients without DM (12.8% vs 11.9%) (**Table 1**).

However, among subjects with DM, those who presented superinfections had a lower BMI (27.4 [22.4-41] vs 30.3 [22.8-8.47], p=0.007), lower HbA1c (6.7 [5.6-8.6] vs. 7.6 [6-12.2] %, p=0.012), and required a higher total daily insulin dose (0.48 [0.15-1.15] vs. 0.58 [0.31-0.93] vs.0.48 [0.15-1.15], p=0.039) than those without superinfections. Regarding to biochemical parameters, patients with superinfections had higher inflammatory parameters than those without superinfections, namely higher CRP (109 [210.6-7232] vs. 60.7 [4.6-212] mg/L, p=0.003), pro-calcitonin (0.2 [0-3.3] vs.0.1 [0-0.5] ng/mL, p=0.008), ferritin (547 [90.8-1914.8] vs. 344 [22-1502.4] ng/mL, p=0.009), IL-6 (547 [90.8-1914.8] vs. 30 [1.6-92.1] pg/mL, p=0.005) and lower albumin (33.8 [26.9-42.4] vs. 36 [29.5-43.1] g/L, p=0.002) serum concentrations. In addition, subjects with superinfections presented higher D-dimer (1071 [210.6-7232] vs. 648 [194-5469.6] ng/mL, p=0.018) and AST levels (35 [13.4-233.2] vs. 25.5 [10.7-96.8] U/L, p=0.021) than those without superinfections. Finally, no differences were found in plasmatic glycemic levels at admission, median CBMG, CV nor grade 1 and grade 2 hypoglycemic episodes between both groups (**Table 3**).

**Table 3:** Baseline clinical characteristics and glycemic parameters between patients with diabetes mellitus related to superinfections.

	Patients who did not have any superinfection	Patients who had any superinfection	P-value
**Patients n(%)**	109 (87.2)	16 (12.8)	
Age (years)*	71 (37-91)	77 (61-89)	0.077
**Sex (F/M), n**	48/61	11/5	0.114
BMI (Kg/m²)*	30.3 (22.8-47)	27.4 (22.4-41)	0.007
**Medical History**			
Diabetes mellitus, n(%)	88 (80.7)	13 (81.3)	1
Cardiovascular disease, n(%)	41 (37.6)	7 (43.8)	0.845
Chronic kidney disease, n(%)	26 (23.9)	6 (38)	0.414
Hypertension, n(%)	73 (67)	14 (88)	0.169
Dyslipidemia, n(%)	65 (59.6)	13 (81.3)	0.164
**Glycemic parameters**			
CBG (mg/dL)*	204 (124-316)	206 (139-226)	0.602
SD of CBG (mg/dL)*	63 (31-104)	70 (39-90)	0.566
HbA1c (%)*	7.6 (6-12.2)	6.7 (5.6-8.6)	0.012
CV (%)*	31 (17.1-46.1)	34.4 (19.2-44)	0.122
Total dose of insulin (units/Kg)*	0.54 (0.2-1)	0.45 (0.1-0.7)	0.126
Grade 1 hypoglycemia (episodes)	12	1	0.856
Grade 2 hypoglycemia (episodes)	12	2	0.286
Biochemical markers*			
Leukocytes count (x10^9^/L)	7 (2.9-13.5)	6.6 (3.1-13)	0.979
Neutrophil count (x10^9^/L)	4.7 (1.8-12)	4.3 (1.7-11.2)	0.932
Lymphocyte count (x10^9^/L)	0.9 (0.3-2.6)	0.9 (0.4-1.9)	0.697
CRP (mg/L)	75.9 (6.3-287.4)	76 (4.4-264.2)	0.765
Pro-calcitonin (ng/mL)	0.1 (0-2.5)	0.2 (0.1-1.2)	0.01
D-Dimer (ng/mL)	771 (194.3-8357.3)	1666.5 (398.6-3795.3)	0.025
Troponin (pg/mL)	8.3 (0.32-119)	10.9 (2.5-186.5)	0.548
Ferritin (ng/mL)	413.5 (24.5-1584.1)	479 (73.3-2014.3)	0.808
IL-6 (pg/mL)	32.5 (1.5-240.7)	44.3 (6.2-1014.8)	0.129
Fibrinogen	712.5 (406.2-1034.1)	644 (345.6-1052.8)	0.607
Albumin (g/L)	35.2 (28.2-43.2)	34 (27.7-40.7)	0.379
AST (U/L)	28 (11-154.5)	24.5 (14.5-80.3)	0.576
ALT (U/L)	26 (8-150.5)	20 (10.1-77.1)	0.167
GGT (U/L)	46 (14.2-544.6)	55.5 (21.9-652)	0.279
Cholesterol (mg/dL)	146.5 (35.7-239.4)	139.5 (70.5-218.3)	0.548
HDL-c (mg/dL)	37.6 (20.4-62.4)	39.1 (19.7-1693.5)	0.72
LDL-c (mg/dL)	88 (30.8-165.6)	68.5 (9.3-141.4)	0.296
Triglycerides (mg/dL)	137 (44.8-378.6)	141 (66-391.9)	0.91
eGFR (ml/min/1.73m^2^)	88 (20.7-90)	69.5 (27.8-126.9)	0.077

*Data are median (IQR95%). BMI, body mass index; CBG, capillary blood glucose; CV, coefficient of variability; TDI, total daily insulin dose; CRP, C-reactive protein; IL-6, interleukin 6; AST, aspartate aminotransferase; ALT, alanine transaminase; GGT, gamma-glutamyl transferase; eGFR, estimated glomerular filtration rate.

## Discussion

Treatment with dexamethasone has been shown to improve prognostic outcomes in subjects infected with COVID-19, especially in severe cases. The impressive results of the RECOVERY trial (13) established that dexamethasone treatment (6 mg daily for 10 days) reduced mortality in hospitalized patients with COVID-19 and respiratory failure who required therapy with supplemental oxygen or mechanical ventilation. Despite 25% of subjects in the RECOVERY trial having DM, this subset of patients was not analyzed separately. Hyperglycemia, a common side effect of corticosteroids that usually requires insulin treatment, is associated with worse outcomes in COVID-19 (2, 3). Therefore, data regarding glycemic control and its relationship with clinical prognosis in subjects with DM treated with the same regimen of dexamethasone as that used in the RECOVERY trial, are needed.

The present study shows that in hospitalized patients with COVID-19 treated with dexamethasone at the same regimen as in the RECOVERY trial, those with DM required more IMV or admission to the ICU and presented a longer LOS than subjects without DM. Also, mortality was higher in people with DM, although differences were not statistically significant, probably due to the lower number of cases. These results are in line with previous studies that showed that mortality risk in subjects with DM and COVID-19 is increased between two and three-fold times compared with subjects without DM (1, 2, 4, 20). As far as we know, this is the first study reporting on the impact of several factors, among them glycemic metabolic control, on the composite outcome of IMV or admission to the ICU in subjects with COVID-19 treated with dexamethasone.

We also found that, in subjects with DM, superinfections are associated with both an increased risk of the composite outcome of IMV or admission to the ICU as well as with a longer LOS. Previous studies suggest that viral respiratory diseases and COVID-19 in particular, may predispose to other fungal, bacterial, and viral co-infections and superinfections (21). On the other hand, corticosteroid therapy has been reported to be associated with an increased risk of infections (22), partly due to its immunosuppressive effects. However, few data exist in the literature regarding superinfections in subjects with COVID-19 during hospital stay. In a recently published study by Garcia-Vidal et al, the prevalence reported of superinfections in subjects admitted with COVID-19 was 4,7% (23) although no data regarding steroid treatment was reported. However, they observed that patients with superinfections had prolonged LOS and higher mortality, similar to the results obtained in the present study.

In addition, our results show that disease severity at admission, reflected by the NEWS2 score within 24 hours of hospital admission, is independently associated with the risk of the composite outcome of IVM or admission to the ICU whereas the inflammatory cytokine IL-6 concentrations are associated with a longer hospital stay. To the best of our knowledge, publications regarding the performance of the NEWS and its modification, NEWS2, in COVID-19 patients are scarce. These scores are validated for the identification of patient deterioration in a range of clinical conditions and settings (24) and the WHO recommends its use in COVID-19 patients (17). Recently, it has been reported that NEWS and NEWS2 are good predictors of death and ICU admission in subjects with COVID-19 (25). A retrospective study involving 334 patients, reported that NEWS was the most accurate predictor of ICU admission (AUROC=0.80 (0.76-0.84)) along with NEWS2 (AUROC=0.78 (0.73-0.82)) within 48h of emergency department arrival (26). Furthermore, in a smaller study, Gidari et al. showed similar AUROC values (AUROC=0.90 (0.82-0.97) for ICU requirement using NEWS2 score at admission (27). Consistently, results obtained in the present study confirm that NEWS2 is an important predictor for the composite outcome of IMV or admission to the ICU in subjects with DM and receiving treatment with dexamethasone.

In subjects with COVID-19, several biomarkers among them IL-6, CRP, ferritin, and low albumin levels have been reported to be predictive of mortality in hospitalized patients with COVID-19 (28). On the other hand, a great amount of data worldwide reveals that COVID-19 patients with hyperglycemia or T2D have a greatly enhanced release of inflammatory cytokines or the cytokine storm syndrome, which leads to immunosuppression and multi-organ failure (29), providing further evidence of a potential link between DM and COVID-19 due to inflammation and immune deficiency. In our study, neither inflammatory markers nor parameters related to glycemic control were found as independent factors associated with the composite outcome of IMV requirement or ICU admission, which may be due to the protective effects of dexamethasone. Regarding LOS, in our study higher IL-6 levels were associated with more days of admission. This finding correlates with the study results of Laguna-Goya R et al. (28) (R²= 0.12; p<0.001). Indeed, inflammatory indices, including LDH, CRP and IL-6 may help to identify cases with dismal prognoses in COVID-19 subjects and perform a prompt intervention in order to improve outcomes (30). Besides inflammatory markers, in our study we found that AST was significantly higher in patients with a longer LOS, IMV requirement or admission to the ICU and mortality. These results are similar to those found in a retrospective multicenter study involving 5,771 patients where AST at admission was strongly associated with mortality risk suggesting liver damage (31).

In this study, no parameters related to glycemic control were associated with the composite outcomes, regardless that prior to the COVID-19 pandemic, DM was associated with increased risk for multiple infections, and infection-related morbidity and mortality often proportional to the degree of glycemic control (32). These results may thus be interpreted as a protective effect of dexamethasone in DM subjects, irrespective of their glycemic control, therefore reinforcing the appropriateness of including this drug in the treatment of patients severely affected by COVID-19 infection and, particularly, in those having DM, a condition that has been recognized as an important risk factor for worse outcomes in such patients, including mortality. Hyperglycemia is a well-known marker of disease severity, and its association with poor outcomes in patients with COVID-19 has been previously reported. Insulin administration is the preferred way to control hyperglycemia in hospitalized patients with a starting threshold below 180 mg/dl (33). This has been described for hyperglycemia at hospital admission (20), for severe hyperglycemia after hospital admission as well as for poorly controlled blood glucose during hospital stay (34). Recent retrospective studies have suggested that insulin levels might be related to death rate in COVID-19 subjects with DM (35). In the present study, insulin doses during LOS were higher in subjects who required IMV or were admitted to the ICU compared with those who did not, reflecting an enhanced insulin-resistance state in these patients, insulin dose was not independently associated with the composite endpoint of IMV or admission to the ICU.

Most analyses reported so far have not accounted for disease severity at the time of hospital admission. In the present study, in our severe cases requiring dexamethasone treatment, parameters related to glycemic control were not associated with IMV or admission to the ICU possibly due to the fact that the inflammatory response was modulated by the corticosteroid therapy which attenuated the deleterious effect of hyperglycemia.

This was a retrospective study and therefore, the studied groups were not strictly matched. Also, the prevalence of DM in our study was higher (59%) compared to the prevalence of inpatients with DM and COVID-19 during the first wave in other Spanish studies (19.4-27%) (20, 36). However, the DM definition used in these studies was more strict. In a Turkish cohort using a similar definition of DM, the prevalence of DM (59.3%) was comparable to the one obtained in our study (37). An additional limitation was that the follow-up time was restricted to hospital stay or death. Although the small size of the study (209 subjects), the main strength of this study treatment was very homogeneous in a single hospital and with the same protocol applied to all of the patients, including the same dose and start time of dexamethasone and the same basal-bolus insulin regimen.

In conclusion, people with DM had about 3 times worse outcomes and IMV/ICU requirements than those without DM while severely infected with COVID-19. Parameters related to glycemic control in these patients in treatment with daily 6 mg of dexamethasone for 10 days were not associated with IMV requirement, admission to ICU, LOS nor mortality. These findings support a positive action on dexamethasone treatment able to attenuate the deleterious effect of hyperglycemia, while nosocomial infections and disease severity at admission were the main factors independently associated with the composite outcome studied (IMV requirement and/or ICU admission).

## Data availability statement

The original contributions presented in the study are included in the article/**Supplementary Material**. Further inquiries can be directed to the corresponding author.

## Ethics statement

The studies involving human participants were reviewed and approved by Ethics Committee of the HUGTiP (approval no: PI-20-175). Written informed consent for participation was not required for this study in accordance with the national legislation and the institutional requirements.

## Author contributions

All authors had full access to all of the data in this study and took complete responsibility for the integrity of the data and the accuracy of the data analysis. All named authors meet the International Committee of Medical Journal Editors (ICMJE) criteria for authorship for this manuscript, take responsibility for the integrity of the work as a whole, and have given final approval for the version to be published.
