# Supplementary material for: Factors associated with critical care requirements in diabetic patients treated with dexamethasone for COVID-19 infection in the first wave of the pandemia
Source: Front Endocrinol (Lausanne). 2022 Dec 22;13:1009028. doi: 10.3389/fendo.2022.1009028 (PMC9815103; doi:10.3389/fendo.2022.1009028)
Supplement: Supplementary file 1 [file Table_1.docx]

Supplementary Material

# Supplementary Data

**HOSPITAL PROTOCOL FOR HYPERGLYCEMIA IN COVID-19 PATIENTS TREATED WITH DEXAMETHASONE**

1. **Non diabetic patients starting treatment with dexamethasone**

| CBGM pre-meals were indicated:   - If BG <180mg/dL: no insulin treatment was indicated - If BG ≥ 180 mg/dl in two consecutive measures: insulin treatment was iniciated |
| --- |
| TDI dose was calculated according to CBGM:   - CBGM 180-250 mg/dl: 0.5 UI/K/d - CBGM 251-300 mg/dl: 0.7 UI/K/d - CBGM >300 mg/dl: 0.9 UI/K/d |
| **40% of total daily dose will be given as glargine in the morning and 60% as aspart pre-meals** |
| **Daily insulin adjustment** |
| Fasting and pre-meal BG between 100-180 mg/dl without hypoglycemia the previous day: same dose |
| Fasting and pre-meal BG >180 mg/dl: increase glargine dose by 10% |
| Fasting and pre-meal BG between 70-99 mg/dl: decrease glargine dose by 10% |
| If hypoglycemia (<70 mg/dL): decrease glargine dose by 20% |
| If hypoglycemia (<40 mg/dL): decrease glargine dose by 30-40%. |
| **Daily aspart insulin adjustment** |
| Increase aspart insulin dose following the scale (supplementary table 3) for BG >140 mg/dl based on TDI dose |
| Decrease aspart insulin dose following the scale (supplementary table 3) for BG <80 mg/dl based on TDI dose |

*****Abbreviations: BG, blood glucose; CBGM, capillary blood glucose measurement; TDI, total daily insulin

1. **Diabetic patients starting treatment with dexamethasone**

| Patients treated with diet or only one oral antidiabetic agent   - TDI: 0.8 U/K/d |
| --- |
| Patients treated with more than one oral antidiabetic agent   - TDI: 1 U/K/d |
| Patients treated with basal insulin +/- aGLP1 +/- any oral antidiabetic   - TDI: 1.2 U/K/d |
| Patients treated with basal-bolus regime +/- any oral antidiabetic   - Increase 50% usual TDI |
| **40% of total daily dose will be given as glargine in the morning and 60% as aspart pre-meal** |
| **Daily glargine insulin adjustment** |
| Fasting and pre-meal BG between 100-180 mg/dl without hypoglycemia the previous day: no change |
| Fasting and pre-meal BG >180 mg/dl: increase glargine dose by 20% |
| Fasting and pre-meal BG between 70-99 mg/dl: decrease glargine dose by 10% |
| If hypoglycemia (BG <70 mg/dL): decrease glargine dose by 20% |
| If hypoglycemia (BG <40 mg/dL): decrease glargine dose by 30-40%. |
| **Daily aspart insulin adjustment** |
| Increase aspart insulin dose following the scale (supplementary table 3) for BG >140 mg/dl based on TDI dose |
| Decrease aspart insulin dose following the scale (supplementary table 3) for BG <80 mg/dl based on TDI dose |

*****Abbreviations: BG, blood glucose; CBG, capillary blood glucose; TDI, total daily insulin

1. **Aspart Insulin Scale**

| **BG (mg/dL)** | **TDI <50 UI** | **TDI 50-100 UI** | **TDI >100 UI** |
| --- | --- | --- | --- |
| - **< 150 mg/dL** | - - | - - | - - |
| - **151-190 mg/dL** | - +2 | - +2 | - +4 |
| - **191-230 mg/dL** | - +3 | - +4 | - +6 |
| - **231-270 mg/dL** | - +4 | - +6 | - +8 |
| - **271-310 mg/dL** | - +6 | - +8 | - +10 |
| - **>310 mg/dL** | - +8 | - +10 | - +12 |

*****Abbreviations: BG, blood glucose; TDI, total daily insulin
